# Vitiligo-Like Hypopigmentation Induced by Ribociclib in a Patient With Metastatic Breast Cancer

**DOI:** 10.7759/cureus.109083

**Published:** 2026-05-18

**Authors:** Israe El Ghazouli, Salim Gallouj, Ouiame El Jouari

**Affiliations:** 1 Dermatology Department, Faculty of Medicine and Pharmacy, Abdelmalek Essaadi University, University Hospital Mohammed VI, Tangier, MAR

**Keywords:** cdk4/6 inhibitors, checkpoint inhibitors, ribociclib, skin toxicities, vitiligo-like

## Abstract

Cyclin-dependent kinase (CDK) 4/6 inhibitors are targeted therapies widely used in the treatment of hormone receptor-positive, human epidermal growth factor receptor 2 (HER2)-negative advanced breast cancer. Although these agents are generally well tolerated, several cutaneous adverse effects have been reported, including rare cases of vitiligo-like depigmentation. Such pigmentary changes may affect treatment adherence and quality of life. Interestingly, emerging evidence suggests a possible association between these immune-mediated skin manifestations and improved survival outcomes. Further studies are needed to better understand the underlying mechanisms, particularly the role of immune pathways, and to determine the potential prognostic significance of these reactions. We describe a 65-year-old woman with metastatic luminal A, HER2-negative breast cancer treated with ribociclib who developed multiple depigmented patches after 12 months of therapy. Lesions were stable and asymptomatic, allowing continuation of ribociclib. Ribociclib-induced vitiligo-like depigmentation is an under-recognized adverse effect. Early recognition and multidisciplinary management are key to optimizing oncologic care without compromising therapeutic benefit.

## Introduction

Cyclin-dependent kinase (CDK) 4/6 inhibitors, including ribociclib, palbociclib, and abemaciclib, have significantly improved survival in patients with hormone receptor-positive, human epidermal growth factor receptor 2 (HER2)-negative advanced breast cancer. While their safety profile is generally favorable, dermatologic side effects remain less studied. Commonly stated side effects include alopecia, pruritus, and xerosis, whereas vitiligo-like hypopigmentation has been reported only rarely. The literature has established a link between treatment-related toxicities and clinical outcomes across several classes of anticancer agents. For instance, immunotherapy-induced, immune-related adverse events are well recognized as predictors of favorable therapeutic response and may be associated with survival benefits [1-3].

Ribociclib, a CDK4/6 inhibitor, is widely used in the treatment of metastatic breast cancer. We report the case of a woman with metastatic breast cancer who developed vitiligo-like lesions after one year of ribociclib therapy. Given the increasing use of this agent and the psychosocial burden associated with vitiligo [4,5], awareness of this potential adverse effect is essential, and appropriate patient counseling should be considered before initiating therapy.

## Case presentation

A 65-year-old woman with a history of breast cancer underwent mastectomy in 2018 for luminal A, HER2-negative disease. After four years, she developed bone and lymph node metastases. Systemic therapy was initiated with ribociclib (400 mg daily, three weeks on/one week off) in combination with anastrozole and zoledronic acid. She demonstrated a positive therapeutic clinical and radiological response, with disease stabilization. Approximately 12 months after starting ribociclib, the patient noticed the appearance of hypopigmented macules on her face, upper limbs, and lower limbs.

**Figure 1:**
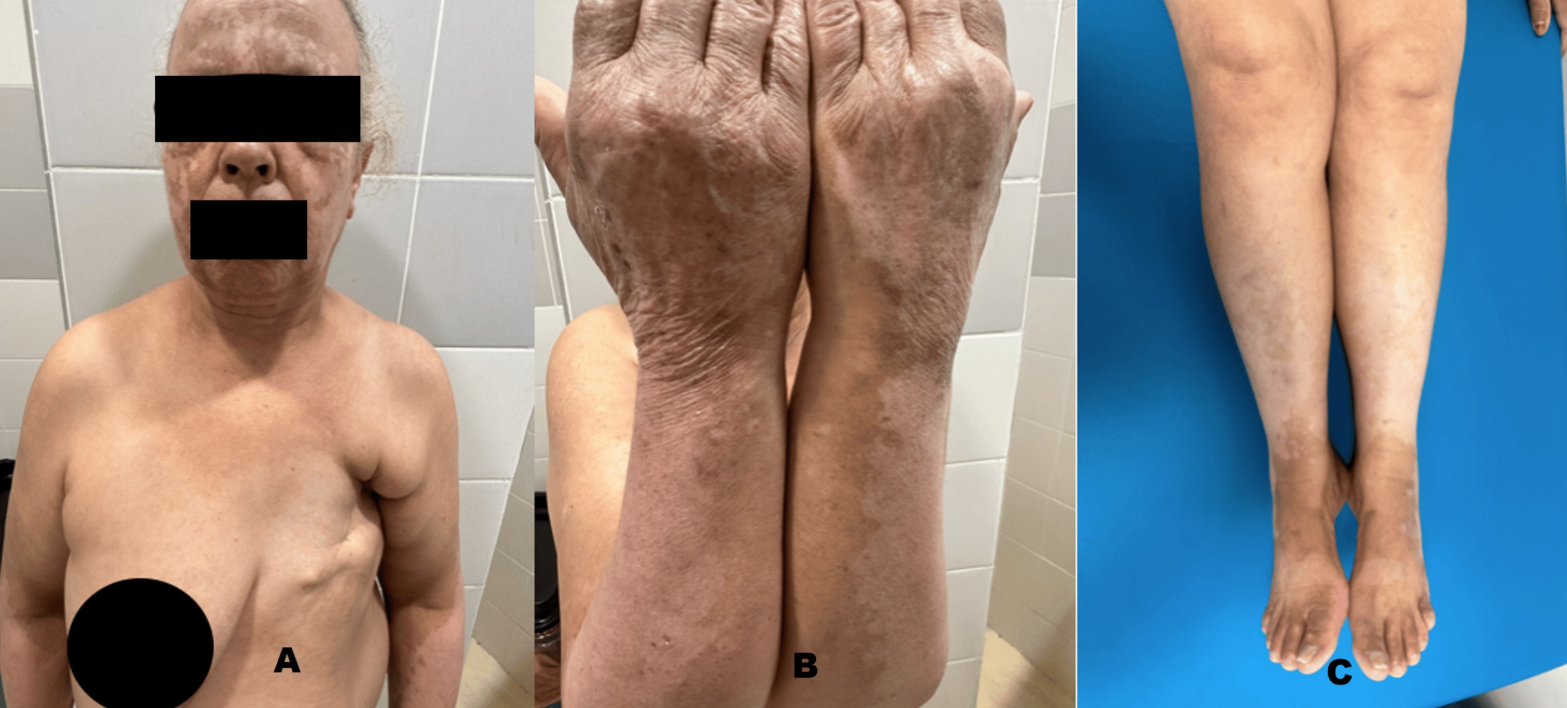
Clinical presentation: Multiple, well-demarcated hypopigmented macules on the patient's face, forearms, hands, and legs without signs of inflammation or scaling, consistent with ribociclib-induced vitiligo-like lesions

**Figure 2:**
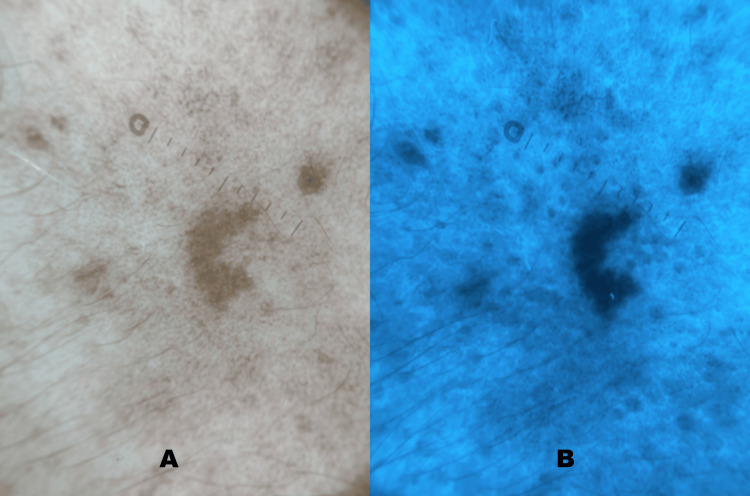
Dermoscopy showing confetti-like depigmentation and ill-defined borders due to pigment loss (Dermlite DL5) UV dermoscopy showing perifolicular pigmentation and an altered pigment network (Dermlite DL5)

**Figure 3:**
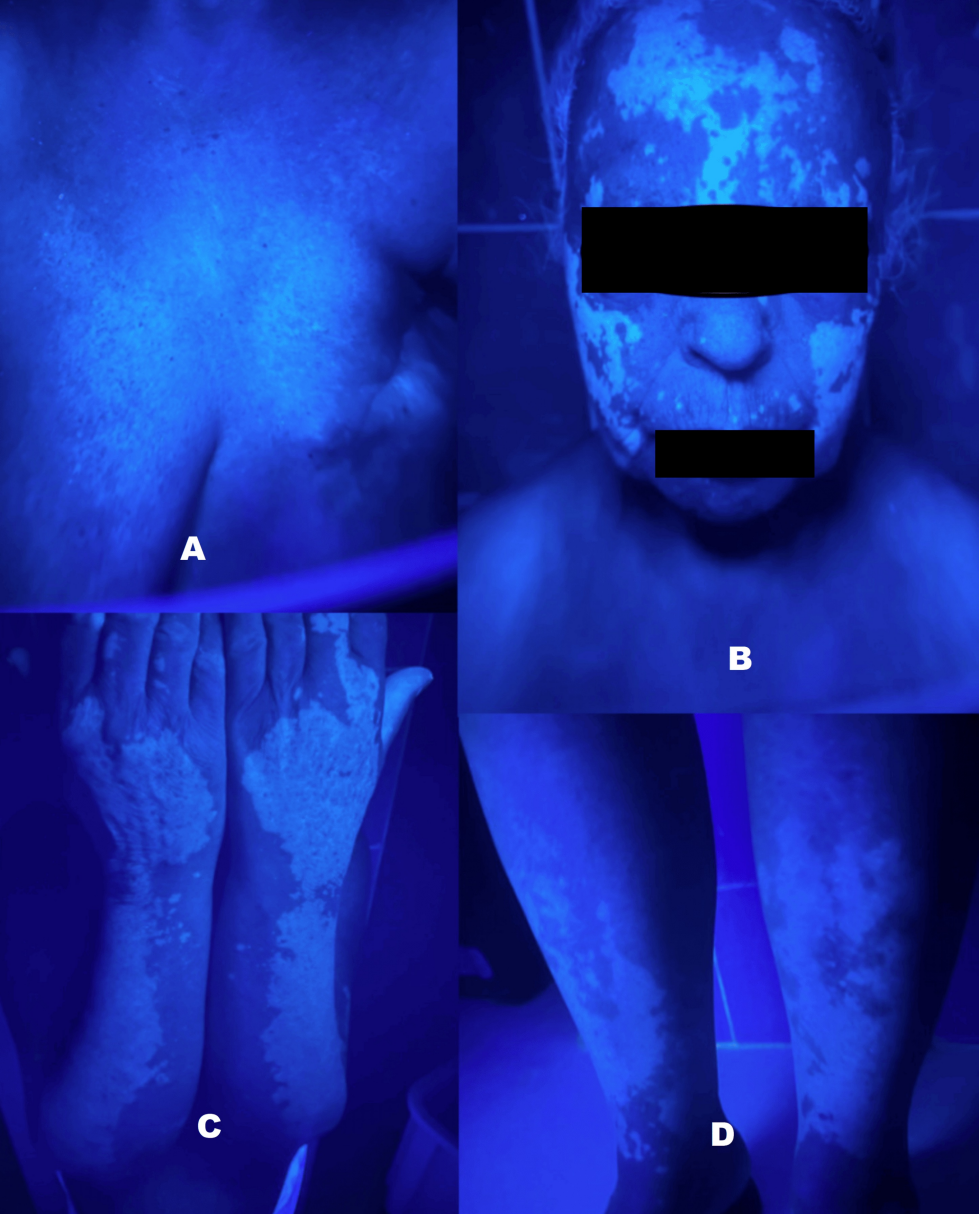
Examination under Wood's lamp, which notably intensified the white color of the lesion

Dermatologic examination revealed multiple well-demarcated, non-scaly hypopigmented patches on both photoexposed and non-photoexposed areas, consistent with a vitiligo-like eruption (Figures 1A-1C). The lesions were asymptomatic, and the patient denied pruritus, burning, or previous similar conditions. Dermoscopy showed confetti-like depigmentation and ill-defined borders, and UV dermoscopy showed perifolicular pigmentation and an altered pigment network, suggesting early activity (Figures 2A-2B). Examination under Wood's lamp showed chalky-white vitiligo-like fluorescence (Figures 3A-3D). Her medical history was negative for autoimmune disorders or a family history of vitiligo. Laboratory studies, including thyroid function and autoantibody screening, were within normal limits (Table 1).

**Table 1:** Screening work-up for other autoimmune disorders commonly associated with vitiligo, including diabetes mellitus and autoimmune thyroiditis

Parameter	Result	Reference Range	Units	Interpretation
Fasting blood glucose	0.9	0.70-1.10	g/L	Within normal limits
Thyroid-stimulating hormone (TSH)	2	0.4-4.0	mIU/L	Within normal limits
Anti-thyroperoxidase antibodies¹	17	< 35	IU/mL	Negative/Normal

No other recent medications were identified. Based on clinical findings and temporal association with ribociclib, a diagnosis of ribociclib-induced vitiligo-like depigmentation was made.

Management included reassurance, photoprotection, and topical corticosteroids. Ribociclib therapy was continued unchanged due to its oncologic efficacy and the mild, non-disabling nature of the cutaneous toxicity. At the six-month follow-up, hypopigmented macules persisted but remained stable without progression. The patient maintained an acceptable quality of life and effective oncologic response.

## Discussion

Vitiligo is an acquired depigmenting disorder caused by autoimmune-mediated destruction of melanocytes, leading to progressive hypopigmented lesions. Although spontaneous vitiligo is relatively common, drug-induced vitiligo-like eruptions are rare and have been increasingly recognized in oncology. These have been described most notably with immune checkpoint inhibitors and tyrosine kinase inhibitors, and more recently with CDK4/6 inhibitors such as ribociclib [6-8].

Among CDK4/6 inhibitors, ribociclib appears more frequently implicated. In a multicenter series, Sollena et al. reported 16 patients with CDK4/6 inhibitor-induced vitiligo-like depigmentation, 14 of whom were on ribociclib. The median onset was approximately 9-12 months after initiation, consistent with the timeline observed in our patient. Lesions were predominantly located on photoexposed areas, though covered sites were also affected [1]. A systematic review by Silvestri et al. further confirmed that dermatologic adverse events, while usually mild, include vitiligo-like eruptions that can impact adherence and quality of life [2].

The exact pathogenesis of ribociclib-induced vitiligo remains unclear. Proposed mechanisms include drug-induced immune dysregulation, unmasking of latent autoimmunity, enhanced antigen presentation, and direct off-target cytotoxicity, leading to melanocyte apoptosis [9]. Interestingly, recent studies suggest that cutaneous immune-mediated toxicities in oncology may correlate with favorable tumor responses, raising the possibility that vitiligo-like reactions could serve as a surrogate marker of therapeutic efficacy [10].

Differential diagnoses include post-inflammatory hypopigmentation and progressive macular hypomelanosis (PMH). Histopathology, when performed, shows melanocyte loss similar to that seen in idiopathic vitiligo, but the clinical context of drug exposure is essential to establishing causality [10].

Management is usually conservative. Most cases can be addressed with topical corticosteroids, calcineurin inhibitors, emollients, and photoprotection (6,8,9). Ribociclib discontinuation or dose adjustment is rarely necessary unless lesions are extensive, symptomatic, or associated with additional severe cutaneous toxicities. Importantly, in our patient, ribociclib was maintained given its favorable therapeutic response, with supportive dermatologic measures only - an approach aligned with recommendations to balance oncologic efficacy with dermatologic safety [6,10].

This case adds to the growing body of evidence identifying vitiligo-like hypopigmentation as a cutaneous adverse effect of ribociclib. Dermatologists play a pivotal role in ensuring timely recognition, patient reassurance, and multidisciplinary management. Further research is warranted to elucidate the immunologic mechanisms underlying this toxicity and to determine whether these reactions have prognostic implications in breast cancer treatment.

## Conclusions

Vitiligo-like hypopigmentation represents a rare, but increasingly recognized, cutaneous adverse effect associated with ribociclib and other CDK4/6 inhibitors. Although the condition is not life-threatening, its appearance can have a significant psychosocial impact on patients and may lead to unnecessary treatment interruptions if not properly identified. Our case emphasizes the importance of maintaining a high index of suspicion for dermatologic toxicities in oncology practice, as early recognition allows for conservative management strategies that preserve both patient quality of life and oncologic efficacy.

Furthermore, this report reinforces the need for multidisciplinary collaboration between oncologists and dermatologists to ensure accurate diagnosis, appropriate counseling, and individualized management. Given the growing use of ribociclib in metastatic breast cancer, further research is warranted to clarify the underlying immune-mediated mechanisms, to explore the potential prognostic significance of this toxicity, and to develop standardized guidelines for its management. Ultimately, integrating dermatologic vigilance into routine oncology care can optimize patient outcomes while safeguarding the therapeutic benefits of CDK4/6 inhibitors.
